# Preliminary fast diagnosis of severe fever with thrombocytopenia syndrome with clinical and epidemiological parameters

**DOI:** 10.1371/journal.pone.0180256

**Published:** 2017-07-05

**Authors:** Jianli Hu, Zhifeng Li, Lei Hong, Changjun Bao, Zhong Zhang, Hongying Zhang, Hao He, Xiaochen Wang, Wendong Liu, Zhihang Peng, Limin Shi, Fengcai Zhu

**Affiliations:** 1 Department of Acute Infectious Disease Control and Prevention, Jiangsu provincial Center for Disease Control and Prevention, Nanjing, China; 2 Medical School, Nanjing University, Nanjing, China; 3 Department of Acute Infectious Disease Control and Prevention, Nanjing Municipal Center for Disease Control and Prevention, Nanjing, China; 4 Department of Disease Control and Prevention, Gulou District Center for Disease Control and Prevention, Nanjing, China; 5 School of Public Health, Nanjing Medical University, Nanjing, China; University of Texas Medical Branch, UNITED STATES

## Abstract

**Objectives:**

To identify specific clinical and epidemiological parameters for clinical diagnosis of SFTSV infection with relatively higher accuracy.

**Methods:**

231 suspected cases of SFTS were reported by various medical institutions from 2011 to 2013 in Jiangsu Province, China. They were followed with SFTSV diagnosis tests and interview-administered questionnaires about demographic characteristics, clinical symptoms and epidemiological exposure factors. Univariate and multivariable logistic regression analysis were used to examine the diagnostic value of these parameters.

**Results:**

SFTSV infection occurred only from April to October annually and usually in hilly areas of specific regions. Three prediction models of SFTSV infection were constructed. Model 3 with clinical and epidemiological parameters combined the benefits of both Model 1and Model 2, which was optimal and had an overall accuracy of 80.2%. Independent indicators for clinical diagnosis of SFTSV infection in Model 3 were as follows: lymphadenopathy (*P* = 0.01), leucopenia (*P*<0.01), age >50 years (*P* = 0.01), tick bites (*P*<0.01), raising domestic animals in the residential areas (*P*<0.01) and farming (*P* = 0.03).

**Conclusions:**

Our results show that using a combination of clinical and epidemiological parameters may be a feasible strategy to provide preliminary fast diagnosis as differentiating SFTSV infection from SFTS-like diseases, thus reducing the risk of misdiagnosis.

## Introduction

Severe fever with thrombocytopenia syndrome (SFTS) is an emerging life-threating infectious disease, which might have emerged as early as 2005 in Jiangsu Province, Anhui Province, and Hubei Province but was first identified to associate with a novel bunyavirus infection in 2009 [1]. The novel bunyavirus belongs to phlebovirus in the family *Bunyaviridae* and is officially named as severe fever with thrombocytopenia syndrome bunyavirus (SFTSV). Ticks, especially *Haemaphysalis longicornis*, are considered as the main vectors [2, 3]. So far more than 2000 cases were reported from at least 16 provinces in China [4]. SFTS cases were confirmed in Japan and Korea in 2013 [5, 6]. In the United States, two SFTS-like cases were identified in Missouri in 2012 which was caused by Heartland virus, a novel *Phlebvirus* which was highly homologous with SFTSV [7]. The case fatality varied geographically and temporally from around 10 percent to about 50 percent. The SFTSV can transmit person to person via contact with blood or bloody cremation of patients, similar to Ebola virus [8–10]. No effective vaccine is available at present. Studies have shown that symptomatic and supportive treatment in an early stage of SFTS can reduce the case fatality greatly [11, 12].

Generally SFTS has an acute onset and the clinical symptoms including high fever, fatigue, myalgia, chilly, headache, lymphadenopathy, gastrointestinal symptoms and hemorrhagic tendency. Leukopenia, thrombocytopenia, and elevated liver-related aminotransferase are the most common abnormalities on laboratory testing [13, 14]. These clinical symptoms of SFTSV infection are nonspecific. Several diseases, including common diseases (e.g., gastrointestinal disease and respiratory disease), leptospirosis, haemorrhagic fever with renal syndrome, and human granulocytic anaplasmosis, share many clinical features with SFTS. It was reported that 30–80% of suspected SFTS cases showed positive for SFTSV by laboratory testing and others were actually misdiagnosis cases of SFTS [15–18].

The keys to disease treatment are the proper employment of effective drugs and supportive treatment under the correct guidance of etiologic diagnosis. An epidemiological data revealed that the majority of SFTS cases came from the backward rural areas [19]. At present, detecting RNA of SFTSV using reverse-transcription polymerase chain reaction (RT-PCR) is the prime etiologic diagnostic method of SFTS, which requires expensive instruments, specialized laboratories and experienced technicians, limiting its use in SFTS endemic areas [20]. Therefore, identification of some clinical and epidemiological parameters, which can provide preliminary fast diagnosis as differentiating SFTSV infection from SFTS-like diseases, would help clinicians to make a suggestive diagnosis. We comparatively analyzed the clinical and epidemiological characteristics of the laboratory-confirmed cases and misdiagnosis cases among 231 suspected SFTS cases, in order to identify indicators for clinical diagnosis of SFTSV infection with relatively higher accurary.

## Methods

### Case definition and classification

In accordance with the national guideline for prevention and control of SFTS issued by the Chinese Ministry of Health, suspected cases are defined as persons who present with acute onset of fever (≥38°C) and other symptoms(e.g., gastrointestinal symptoms, bleeding), epidemiological exposure factors (e.g., being exposed to ticks 2 weeks before illness onset) and laboratory data showing thrombocytopenia or leukopenia. Laboratory-confirmed cases are defined as suspected cases who meet one or more of the following criteria: (1) detection of SFTSV RNA, (2) seroconversion or ≥4-fold increase in IgG against SFTSV between two serum samples collected at least 2 weeks apart, and (3) isolation of SFTSV. Others of the suspected cases are defined as misdiagnosis cases of SFTS.

### Sample collection and epidemiological investigation

When suspected cases were detected by medical institutions, the serum samples were collected and sent to Jiangsu Provincial Center for Diseases Control and Prevention (CDC) for testing within 24 hours. At the same time, an epidemiological investigation was initiated. A standardized questionnaire including demographic characteristics, clinical symptoms and epidemiological exposure factors (living and working environment, and exposure history within the previous 2 weeks prior to illness onset) was used (S1 Appendix). Professionals from local CDC interviewed suspected cases, their family members and the clinicians in charge. Additionally, all cases’ medical records were reviewed one week after their discharges or deaths. To further analyze the indicators for clinical diagnosis of SFTSV infection, the suspected cases were categorized into laboratory-confirmed cases and misdiagnosis cases according to results of laboratory testing.

### Ethical consideration

The study was approved by the Ethics Committee of the Jiangsu Provincial CDC. All aspects of the study complied with the Declaration of Helsinki. The Ethics Committee specifically approved verbal informed consent as an alternate to written informed consent, because data were planned to be analyzed anonymously. Moreover, a small amount of blood samples were acquired from human clinical samples, which were collected by following medical institutions’ approved procedures and only used for laboratory diagnosis of SFTSV infection.

### Statistical analysis

Data were double entered into an EpiData 3.1 (the EpiData Association, Denmark, Europe), and the database consistency check was performed. SPSS version 18.0 (Statistical Product and Service Solutions, Chicago, IL, USA) was used for all statistical analyses. Statistical tests were two-sided with a significance level of 0.05. Chi-square or Fisher’s exact test was used to compare dichotomous variables. Indicators for clinical diagnosis of SFTSV infection were identified using univariate and multivariable non-conditional logistic regression models. The multivariate non-conditional logistic regression models were constructed and included univariate analysis of variables with significance level of ≤0.05. The forward stepwise elimination procedure was applied to exclude the variables with *P*>0.10 in the multivariable model. Consequently, we presented odds ratios (ORs) with 95% confidence intervals (CIs) for various indicators.

## Results

### The spatial and temporal distribution

A total of 231 suspected cases of SFTS were reported by medical institutions from 2011 to 2013 in Jiangsu Province, China. Over 85% suspected cases were reported by clinicians from tertiary hospitals. The average period from illness onset to confirmation of SFTSV infection was 8.5 days, ranging from 1 to 50 days. Of 231 suspected cases, 89 (38.5%) were positive for SFTSV, and 19 died. The case fatality rate was 21.3% (19/89).

**Fig 1:**
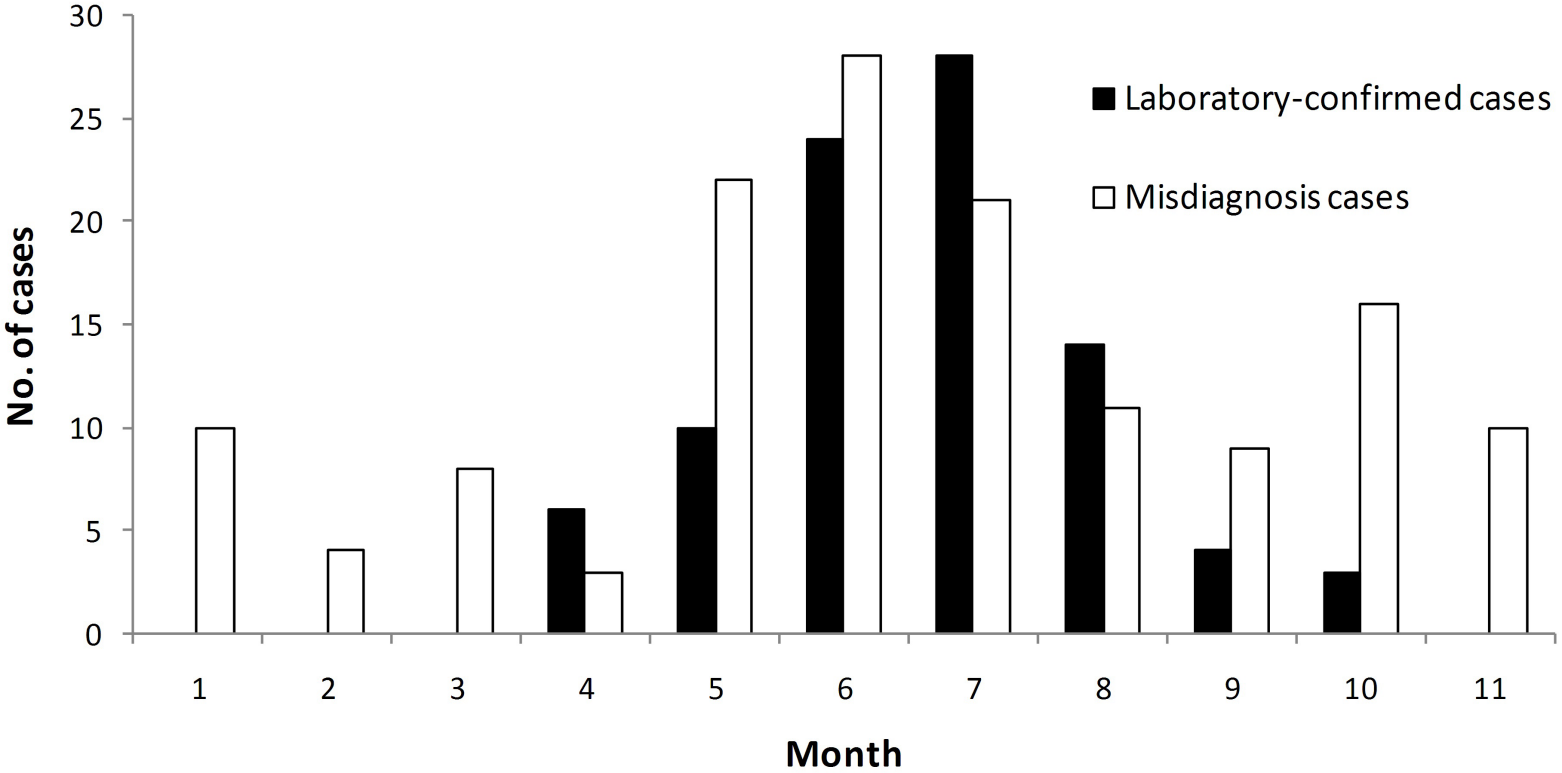
The temporal distribution of laboratory-confirmed cases with SFTSV infection and misdiagnosis cases in Jiangsu Province, China, 2011–2013.

Fig 1 showed that all laboratory-confirmed cases were reported from April to October annually. The incidence peak occurred around July. No cases were detected from November to next March. On the contrary, misdiagnosis cases occurred year-round although a relative peak occurred from May to July.

**Fig 2:**
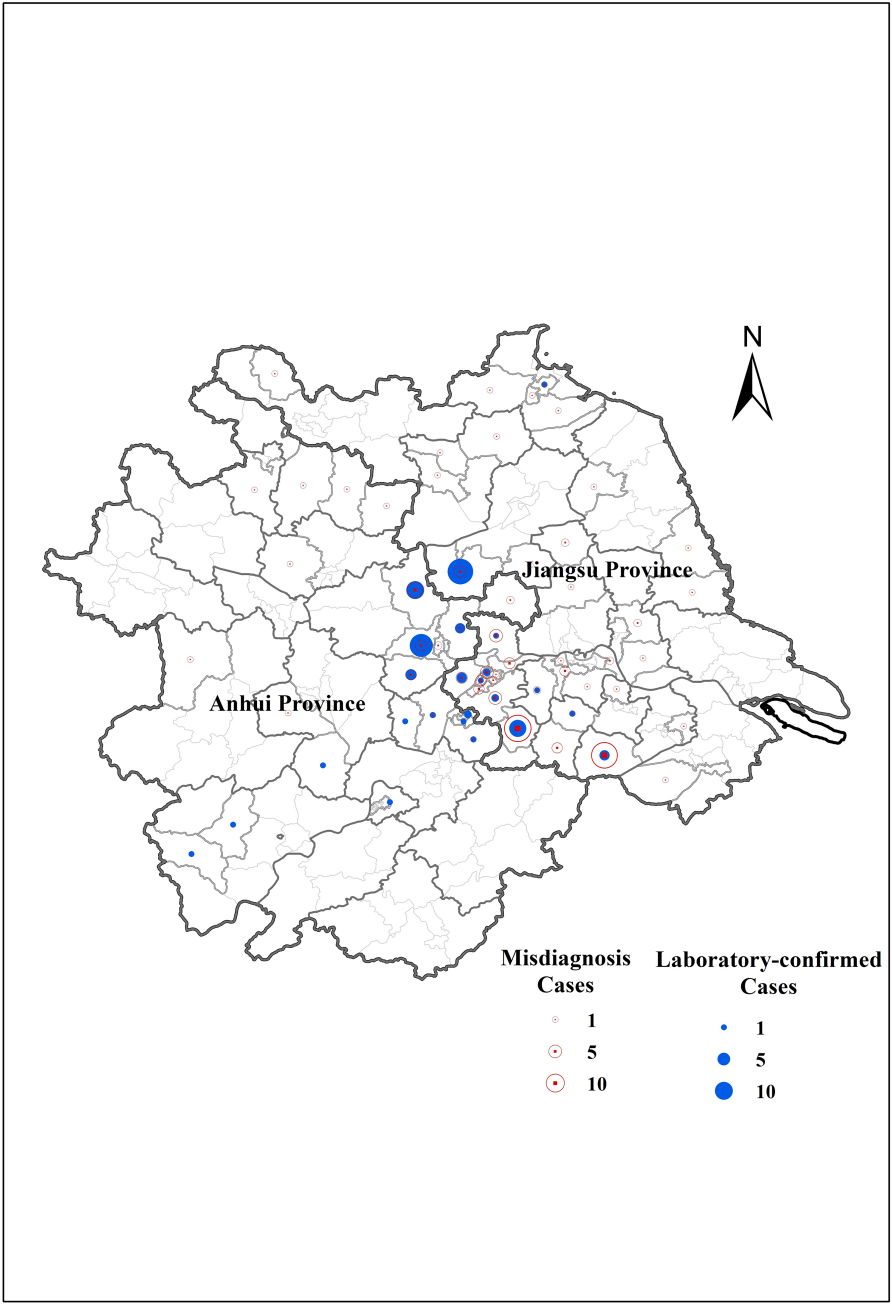
The spatial distribution of laboratory-confirmed cases with SFTSV infection and misdiagnosis cases in Jiangsu Province, China, 2011–2013. The figure was generated using ArcGIS software version 9.3 (ESRI, Redlands, CA, USA). The blue circle denotes the laboratory-confirmed cases, and the red circle denotes the misdiagnosis cases, where the size of the circle means the approximate number. The bold line is the boundary of Jiangsu Province (Up right) and Anhui Province (Down left).

For the geographical distribution, laboratory-confirmed cases were concentrated in hilly areas around the boundary of Anhui Province and Jiangsu Province, while misdiagnosis cases were scattered (Fig 2).

### Demographic and clinical characteristics of SFTSV infection

All of suspected cases ranged from 16 years old to 83 years old and the median age of them were 56.5 years old. 55.8% of them were farmers. In order to facilitate the analysis, we divided suspected cases into two categories by age: >50 years and ≤50 years, and two categories by occupation: farmers or non-farmers. For laboratory-confirmed cases and misdiagnosis cases, respectively, 75.3% and 52.8% were older than 50 years; 70.8% and 46.5% were farmers; 56.2% and 63.4% were male. Univariate analysis indicated that, there were significant differences in age and occupation between two groups (Table 1).

**Table 1:** Demographic and clinical parameters of laboratory-confirmed cases and misdiagnosis cases in Jiangsu Province, China, 2011–2013.

Demographic and clinical parameters	Laboratory-confirmed cases	Misdiagnosis cases	Univariate analysis	Multivariate analysis (Model 1)
	n/N(%)	n/N(%)	*χ*^2^ (P value)	OR(95%CI)
**Demographic parameters**				
Gender(male)	50/89(56.18)	90/142(63.38)	1.18(0.28)	
Age (>50 years)	67/89(75.28)	75/142(52.82)	11.66(<0.01)	2.46(1.24–4.85)
Occupation (farmer)	63/89(70.79)	66/142(46.48)	13.11(<0.01)	1.93(1.01–3.73)
Clinical parameters*				
Fever	89/89(100.0)	139/142(97.9)	0.01(0.95)	
Chilly	47/89(52.8)	95/142(66.9)	4.59(0.03)	
Fatigue	81/89(91.0)	122/142(85.9)	1.33(0.25)	
Headache	38/89(42.7)	75/142(52.8)	2.24(0.13)	
Myalgia	53/89(59.6)	77/142(54.2)	0.63(0.43)	
Nephralgia	10/89(11.2)	19/142(13.4)	0.23(0.63)	
Lymphadenopathy	31/89(34.8)	23/142(16.2)	10.61(<0.01)	3.21(1.57–6.55)
***Gastrointestinal symptoms***				
Anorexia	83/89(93.3)	127/142(89.4)	0.97(0.33)	
Nausea	45/89(50.6)	57/142(40.1)	2.41(0.12)	
Vomiting	39/89(43.8)	45/142(31.7)	3.48(0.06)	
Abdominal pain	24/89(27.0)	38/142(26.8)	0.00(0.97)	
Abdominal distension	21/89(23.6)	35/142(24.6)	0.03(0.86)	
Diarrhea	39/89(43.8)	41/142(28.9)	5.40(0.02)	
***Hemorrhagic tendency***				
Conjunctival congestion	14/89(15.7)	22/142(15.5)	0.00(0.96)	
Skin petechiae	14/89(15.7)	25/142(17.6)	0.14(0.71)	
Gum bleed	6/89(6.7)	10/142(7.0)	0.01(0.93)	
Haematemesis	4/89(4.5)	5/142(3.5)	0.14(0.71)	
***Blood routine examination***				
Leukopenia	70/89(81.4)	67/142(48.6)	24.06(<0.01)	5.17(2.60–10.26)
Thrombocytopenia	78/89(91.8)	116/142(84.7)	2.39(0.12)	

*: The first survey of the clinical parameters was within 24 hours after every suspected case was detected by medical institutions. Leukopenia and thrombocytopenia were determined according to the nadir measurements of every case from illness onset to the first survey.

Symptoms most commonly reported in laboratory-confirmed cases were fever (100.0%), anorexia (93.3%), fatigue (91.0%), and thrombocytopenia (91.8%). In addition, myalgia (59.6%), headache (42.7%), the gastrointestinal symptoms like nausea (50.6%), vomiting (43.8%), and diarrhea (43.8%), superficial lymphadenopathy (34.8%) were more common clinical symptoms. But hemorrhagic tendency like conjunctival congestion (15.7%), skin petechiae (15.7%), gum bleed (6.7%) and haematemesis (4.5%) were relatively rare clinical symptoms.

By univariate analysis, laboratory-confirmed SFTS cases presented clinical manifestations such as diarrhea (*P* = 0.02), lymphadenopathy (*P*<0.01) and leukopenia (*P*<0.01). In contrast, misdiagnosis cases presented “chilly” more frequently (*P* = 0.03). In addition, the highest body temperatures (39.1±0.7°C) during course in the laboratory-confirmed cases were lower than that (39.3±0.8°C) in misdiagnosis cases significantly (*P* = 0.01). There were no significant differences in other clinical symptoms between laboratory-confirmed cases and misdiagnosis cases (Table 1).

### Epidemiological exposure factors of SFTSV infection

For diagnosis of SFTS as a tick-borne zoonosis, the exposure history within two weeks prior illness onset is certainly important. By univariate analysis, exposure to ticks (*P*<0.01), tick bites (*P*<0.01), presence of ticks in the residential areas or workplace (*P*<0.01), tea-picking (*P*<0.01), grazing (*P*<0.01), farming (*P*<0.01), raising domestic animals in the residential areas (*P*<0.01), mowing (*P*<0.01), presence of rats (*P*<0.01) and contact with wild animals (*P* = 0.04) were correlated to laboratory diagnosis with SFTSV infection (Table 2).

**Table 2:** Epidemiological exposure factors of laboratory-confirmed cases and misdiagnosis cases in Jiangsu Province, China, 2011–2013.

Epidemiological exposure factors within two weeks prior illness onset	Laboratory-confirmed cases	Misdiagnosis cases	Univariate analysis	Multivariate analysis (Model 2)
	n/N(%)	n/N(%)	*χ*^2^ (P value)	OR(95%CI)
Farming	65/85(76.47)	44/138(31.88)	41.85(<0.01)	3.85(1.84–8.04)
Mowing	42/85(49.41)	21/137(15.33)	29.98(<0.01)	
Hunting	1/85(1.18)	0/137(98.82)	1.62(0.20)	
Tea-picking	13/85(15.29)	3/137(2.19)	13.47(<0.01)	
Grazing	12/85(14.12)	3/137(2.19)	11.85(<0.01)	
Deforesting	10/85(11.76)	7/137(5.11)	3.29(0.07)	
Traveling to endemic areas	4/85(4.71)	13/135(9.63)	1.77(0.18)	
Raising domestic animals in the residential areas	69/84(82.14)	58/139(41.73)	34.89(<0.01)	3.27(1.43–7.49)
Contact with wild animals	8/82(9.76)	4/134(2.99)	4.45(0.04)	
Presence of ticks in the residential areas or workplace	40/85(47.06)	14/142(9.86)	40.59(<0.01)	2.48(0.98–6.30)
Exposure to ticks	35/84(41.67)	9/142(6.34)	42.02(<0.01)	
Tick bites	21/85(24.71)	5/142(3.52)	23.53(<0.01)	4.35(1.05–17.90)
Presence of rats	53/83(63.86)	45/136(49.45)	19.73(<0.01)	
Non-intact skin	11/82(13.41)	13/133(9.77)	0.68(0.41)	

### Prediction model of SFTS etiology diagnosis

Firstly, with demographic and clinical parameters of *P*≤0.05 by univariate analysis, a multivariable logistic-regression analysis showed that age >50 years (*P* = 0.01), being a farmer (*P* = 0.05), lymphadenopathy (*P*<0.01) and leucopenia (*P*<0.01) were independently associated with SFTSV infection (Table 1). This model (Model 1) showed a sensitivity of 55.8% and specificity of 81.2%, with an overall accuracy of 71.4%. The probability predicted for SFTS etiology was obtained from the following Eq (1):

1/{1+exp[5.903−(0.898×age>50 years)−(0.659×farmer)−(1.165×lymphadenopathy)−(1.642×leucopenia) ] } (1)

Then, only with risk factors of *P*≤0.05 by univariate analysis, a multivariable logistic-regression analysis showed that farming (*P*<0.01), raising domestic animals in the residential areas (*P*<0.01), presence of ticks in the residential areas or workplace (*P* = 0.06) and tick bites (*P* = 0.04) were independently associated with SFTSV infection (Table 2). This model (Model 2) showed a sensitivity of 76.3% and specificity of 78.1%, with an overall accuracy of 77.4%. The probability predicted for SFTS etiology was obtained from the following Eq (2):

1/{1+exp[7.480−(0.910×presence of ticks)−(1.469×tick bites)−(1.186×raising domestic animals)−(1.347×farming)]} (2)

Finally, with demographic parameters, clinical parameters and epidemiological exposure factors, a multivariable logistic-regression analysis showed that lymphadenopathy (*P* = 0.01), leucopenia (*P*<0.01), age >50 years (*P* = 0.01), tick bites (*P*<0.01), raising domestic animals in the residential areas (*P*<0.01) and farming (*P* = 0.03) were independently associated with SFTSV infection (Table 3). This model (Model 3) showed a sensitivity of 68.8% and specificity of 87.1%, with an overall accuracy of 80.2%. The probability predicted for SFTS etiology was obtained from the following Eq (3):

$$\begin{array}{l} 1/\{1+exp[11.816-\left(1.071\times lymphadenopathy\right)-\left(1.124\times leucopenia\right)-\\ \left(1.048\times age>50\;years\right)-\left(2.369\times tick\;bites\right)-\\ \left(1.217\times raising\;domestic\;animals\right)-\left(0.919\times farming\right)]\} \end{array}$$ (3)

**Table 3:** Prediction model of SFTSV infection by combining demographic, clinical and epidemiological parameters (Model 3).

Parameters	β value	S.E	*χ*^2^	P value	OR	95%CI
Tick bites	2.37	0.66	12.97	<0.01	10.68	2.94–38.78
Raising domestic animals in the residential areas	1.22	0.42	8.26	<0.01	3.38	1.47–7.74
Leukopenia	1.12	0.39	8.36	<0.01	3.08	1.43–6.59
Lymphadenopathy	1.07	0.44	5.98	0.01	2.92	1.24–6.89
Age (>50 years)	1.05	0.41	6.41	0.01	2.85	1.27–6.42
Farming	0.92	0.41	5.06	0.03	2.51	1.13–5.58
Consant	11.82	1.94	36.98	<0.01		

## Discussion

SFTS is an emerging tick-borne infectious disease, which has caused comprehensive public health concerns due to its expanding epidemic areas, the capability of human-to-human transmission and a high case fatality rate [4]. The clinical symptoms of SFTS are nonspecific and consistent with many infectious pathogens including bacteria and viruses. It should be mentioned that *Anaplasma phagocytophilum* was considered to be the causative agent of SFTS in China before the discovery of SFTSV in 2010, because patients with human granulocytic anaplasmos is also presented fever, thrombocytopenia and leucopenia [1, 15]. In fact, in most cases viral laboratory confirmation might not always be available due to unaffordability of test kits, lack of instruments and shortage of trained laboratory technicians in rural areas. At present, SFTS misdiagnosis is very common, posing a challenge both to clinicians and public health officials. On the one hand, our findings also disclosed the problem of SFTS misdiagnosis, that 38.5% of 231 suspected SFTS cases reported by medical institutions in Jiangsu Province from 2011 to 2013, were positive for SFTSV. On the other hand, the median time from illness onset to confirmation was 8.5 days in our study, which suggested that clinicians fail to make the timely diagnosis at present. Therefore, we summarized the first attempt to identify clinical and epidemiological parameters as useful indicators for clinical diagnosis of SFTSV infection, which could provide preliminary fast diagnosis as differentiating SFTSV infection from SFTS-like diseases, thus reduce the risk of misdiagnosis.

As a natural foci disease, SFTSV is maintained in a special ecosystem that is best suited to a natural transmission cycle involving tick vectors and animal reservoir hosts. Natural foci can change under climate, geographical and human influence, but they are stable with a long-time activity [21]. From the results, we can conclude that hilly areas around the boundary of Anhui and Jiangsu Province were the potential natural foci of SFTSV. For the temporal distribution, cases with SFTSV infection occurred only from late April to October, which were in agreement with corresponding data from other studies in China, consistent with the life cycle of ticks. Therefore, even if one patient with SFTS-like syndromes was reported from November to next March, we must think carefully diagnosis of SFTS without laboratory testing.

In our study, we constructed three prediction models. Model 1 was based on the demographic and clinical parameters had a higher specificity than Model 2, while Model 2 was only based on the epidemiological exposure factors had a higher sensitivity than Model 1. However, Model 3 which combined the benefits of both Model 1 and Model 2 was optimal and had a highest overall accuracy. At present, the diagnosis of SFTSV infection is often directly derived from clinical characteristics of patients, then pathogenic detection. However, our findings suggest that the epidemiological information of patients plays an important role in SFTS diagnosis, and the clinicians shouldn’t over-depend on pathogenic detection, neglecting the inquiry of epidemiological information after learning about clinical syndromes of patients.

Our study indicated that persons older than 50 years were readily infected with SFTSV, and was consistent with previous conclusion [22]. The high proportion of elderly patients may be due to physiological factors related to aging, such as decreased immune function. Previous study showed that prevalence of antibodies against SFTSV increased with age [22, 23]. However, this may also be related to demographic features of residents in endemic rural areas. Generally, young adults from wooded or hilly areas go to cities to earn better money and the actual residents are just elderly people and children.

Interestingly, cases with SFTSV infection presented some special clinical features. For instance, laboratory-confirmed cases developed more likely gastrointestinal symptoms. In particular, the proportion of diarrhea among laboratory-confirmed cases was significantly higher than that of misdiagnosis cases. But hemorrhagic tendency showed no difference between the two groups. In addition, confirmed cases were more likely to present lymphadenopathy and leucopenia, which became major indexes of the SFTS etiologic diagnosis and retained in the Model 3. Reproduction of virus happened first in neighboring lymph nodes after invasion into the host, which might result in lymphadenopathy. The animal model demonstrated that SFTSV then went into the spleen and liver in which white blood cells were devoured by macrophages [24]. But our study showed that the count of platelet had no significant difference between laboratory-confirmed and misdiagnosis cases although platelets were also devoured in the process.

In our study, epidemiological exposure factors such as tick bites, farming outside two weeks before illness onset and raising domestic animals in the residential areas were helpful to distinguish SFTSV infection from SFTS-like diseases, which were also proved to be risk factors for SFTS in other researches. Ticks, particularly *Haemaphysalis longicornis*, were thought to be the primary vector for SFTSV. First, a high proportion of patients diagnosed with SFTS reported a history of tick bite [1, 25–28]. Second, spatial and temporal distributions of human cases were consistent with the fluctuation of certain species of ticks in a given endemic area [1, 25, 26, 29, 30].Third, several research groups had detected SFTSV-specific nucleotide sequences, or isolated virus from ticks collected from animals, or the environment, which had high homology (93–100%) with SFTSV isolated from patients [1, 2, 16, 25, 29, 31, 32]. Finally, ticks could acquire the virus by feeding on blood of the SFTSV-infected host animal and transstadially and transovarially transmit it to other developmental stages of ticks [2, 3]. Farming activities became one index of the SFTS etiologic diagnosis. The reason for this maybe that SFTS is endemic in rural areas and farming activities around weeds and shrubs increase the exposure opportunities for ticks. It is notable that 82% of laboratory-confirmed cases in our study bred domestic animals before illness onset. We postulated that domestic animals living in close contact with humans were reservoirs for SFTSV, whereas ticks as the vector could transmit the virus from animal hosts to human. Some studies concluded that domestic animals, especially free-range goats, cattle and dogs, had a high seroprevalence of antibodies against SFTVS and confirmed this hypothesis indirectly [23, 33, 34].

One strength of our study was that the median time between suspected diagnosis and information collection for demographic, clinical and epidemiological parameters was less than one hour. Therefore, suggestive diagnosis offered by these parameters would be available much earlier than laboratory tests. Another strength was that using a combination of these parameters may be an only feasible strategy to improve SFTS diagnosis in resource-poor setting, such as rural areas in China, where viral laboratory confirmation might not be available due to no highly specialized laboratories, no trained laboratory technicians or expensive PCR machines.

There were three major flaws of this study. Not all clinical and epidemiological parameters were considered into our study due to difficulty in data collection. For instance, serum abnormal findings such as elevated liver-associated transferase and prolonged activated partial thromboplastin time could be found in majority cases with SFTSV infection. We don’t know whether these abnormities could be used to distinguish SFTSV infection from other pathogen infection. In the future, other clinical and epidemiological parameters related to SFTSV infection will be furtherly identified. Secondly, although study design was perspective, clinical and epidemiological parameters were obtained mainly by interviewing suspected cases, their family members and the clinicians in charge. So there might be memory bias. Thirdly, univariate analysis showed the highest body temperatures (39.1±0.7°C) in the laboratory-confirmed cases were lower than that (39.3±0.8°C) in misdiagnosis cases (*P* = 0.01). From the biological significance, the difference was not significant. Maybe it’s related the small sample size.

Our findings suggest that specific clinical and epidemiological parameters may be useful indicators to assist clinicians to make preliminary fast diagnosis as differentiate SFTSV infection from SFTS-like diseases, thus reduce the risk of misdiagnosis.

## Supporting information

S1 Appendix The questionnaire of suspected cases with SFTSV infection. (DOC)

S1 Table Data file for demographic characteristics, clinical symptoms and epidemiological exposure factors for SFTSV infection of laboratory-confirmed cases and misdiagnosis cases. (XLS)

## References

[1] Yu XJ, Liang MF, Zhang SY, Liu Y, Li JD, Sun YL, et al Fever with thrombocytopenia associated with a novel bunyavirus in China. The New England journal of medicine. 2011;364(16):1523–32. Epub 2011/03/18. doi: 10.1056/NEJMoa1010095 . 21410387 10.1056/NEJMoa1010095 PMC3113718

[2] Wang S, Li J, Niu G, Wang X, Ding S, Jiang X, et al SFTS virus in ticks in an endemic area of China. The American journal of tropical medicine and hygiene. 2015;92(4):684–9. Epub 2015/02/26. doi: 10.4269/ajtmh.14-0008 . 25711611 10.4269/ajtmh.14-0008 PMC4385759

[3] Luo LM, Zhao L, Wen HL, Zhang ZT, Liu JW, Fang LZ, et al Haemaphysalis longicornis Ticks as Reservoir and Vector of Severe Fever with Thrombocytopenia Syndrome Virus in China. Emerging infectious diseases. 2015;21(10):1770–6. Epub 2015/09/25. doi: 10.3201/eid2110.150126 . 26402039 10.3201/eid2110.150126 PMC4593435

[4] Liu K, Zhou H, Sun RX, Yao HW, Li Y, Wang LP, et al A national assessment of the epidemiology of severe fever with thrombocytopenia syndrome, China. Scientific reports. 2015;5:9679 Epub 2015/04/24. doi: 10.1038/srep09679 . 25902910 10.1038/srep09679 PMC4407178

[5] Takahashi T, Maeda K, Suzuki T, Ishido A, Shigeoka T, Tominaga T, et al The first identification and retrospective study of Severe Fever with Thrombocytopenia Syndrome in Japan. The Journal of infectious diseases. 2014;209(6):816–27. Epub 2013/11/16. doi: 10.1093/infdis/jit603 . 24231186 10.1093/infdis/jit603 PMC7107388

[6] Park SW, Han MG, Yun SM, Park C, Lee WJ, Ryou J. Severe fever with thrombocytopenia syndrome virus, South Korea, 2013. Emerging infectious diseases. 2014;20(11):1880–2. Epub 2014/10/24. doi: 10.3201/eid2011.140888 . 25341085 10.3201/eid2011.140888 PMC4214315

[7] McMullan LK, Folk SM, Kelly AJ, MacNeil A, Goldsmith CS, Metcalfe MG, et al A new phlebovirus associated with severe febrile illness in Missouri. The New England journal of medicine. 2012;367(9):834–41. Epub 2012/08/31. doi: 10.1056/NEJMoa1203378 . 22931317 10.1056/NEJMoa1203378

[8] Bao CJ, Guo XL, Qi X, Hu JL, Zhou MH, Varma JK, et al A family cluster of infections by a newly recognized bunyavirus in eastern China, 2007: further evidence of person-to-person transmission. Clinical infectious diseases: an official publication of the Infectious Diseases Society of America. 2011;53(12):1208–14. Epub 2011/10/27. doi: 10.1093/cid/cir732 . 22028437 10.1093/cid/cir732

[9] Liu Y, Li Q, Hu W, Wu J, Wang Y, Mei L, et al Person-to-person transmission of severe fever with thrombocytopenia syndrome virus. Vector Borne Zoonotic Dis. 2012;12(2):156–60. Epub 2011/10/01. doi: 10.1089/vbz.2011.0758 . 21955213 10.1089/vbz.2011.0758

[10] Tang X, Wu W, Wang H, Du Y, Liu L, Kang K, et al Human-to-human transmission of severe fever with thrombocytopenia syndrome bunyavirus through contact with infectious blood. The Journal of infectious diseases. 2013;207(5):736–9. Epub 2012/12/12. doi: 10.1093/infdis/jis748 . 23225899 10.1093/infdis/jis748

[11] Shimojima M, Fukushi S, Tani H, Taniguchi S, Fukuma A, Saijo M. Combination effects of ribavirin and interferons on severe fever with thrombocytopenia syndrome virus infection. Virology journal. 2015;12:181 Epub 2015/11/04. doi: 10.1186/s12985-015-0412-3 . 26527529 10.1186/s12985-015-0412-3 PMC4630909

[12] Shimada S, Posadas-Herrera G, Aoki K, Morita K, Hayasaka D. Therapeutic effect of post-exposure treatment with antiserum on severe fever with thrombocytopenia syndrome (SFTS) in a mouse model of SFTS virus infection. Virology. 2015;482:19–27. Epub 2015/03/31. doi: 10.1016/j.virol.2015.03.010 . 25817401 10.1016/j.virol.2015.03.010 PMC7125729

[13] Gai ZT, Zhang Y, Liang MF, Jin C, Zhang S, Zhu CB, et al Clinical progress and risk factors for death in severe fever with thrombocytopenia syndrome patients. The Journal of infectious diseases. 2012;206(7):1095–102. Epub 2012/08/02. doi: 10.1093/infdis/jis472 . 22850122 10.1093/infdis/jis472

[14] Lu QB, Yang ZD, Wang LY, Qin SL, Cui N, Wang HY, et al Discrimination of novel bunyavirus infection using routine laboratory test. Clinical microbiology and infection: the official publication of the European Society of Clinical Microbiology and Infectious Diseases. 2015;21(2):204.e1–7. Epub 2015/02/07. doi: 10.1016/j.cmi.2014.07.012 . 25658566 10.1016/j.cmi.2014.07.012

[15] Cui F, Cao HX, Wang L, Zhang SF, Ding SJ, Yu XJ, et al Clinical and epidemiological study on severe fever with thrombocytopenia syndrome in Yiyuan County, Shandong Province, China. The American journal of tropical medicine and hygiene. 2013;88(3):510–2. Epub 2013/01/23. doi: 10.4269/ajtmh.11-0760 . 23339197 10.4269/ajtmh.11-0760 PMC3592533

[16] Liu L, Guan XH, Xing XS, Shen XF, Xu JQ, Yue JL, et al [Epidemiologic analysis on severe fever with thrombocytopenia syndrome in Hubei province, 2010]. Zhonghua liu xing bing xue za zhi = Zhonghua liuxingbingxue zazhi. 2012;33(2):168–72. Epub 2012/05/12. . 22575136

[17] Li Y, Zhou H, Mu D, Yin W, Yu H. [Epidemiological analysis on severe fever with thrombocytopenia syndrome under the national surveillance data from 2011 to 2014, China]. Zhonghua liu xing bing xue za zhi = Zhonghua liuxingbingxue zazhi. 2015;36(6):598–602. Epub 2015/11/14. . 26564632

[18] Xie YT, Lai DH, Liu GY, Zhou JL, Lun ZR. Severe fever with thrombocytopenia syndrome in China. Lancet Infect Dis. 2015;15(2):145 Epub 2015/03/10. doi: 10.1016/S1473-3099(14)70891-6 . 25749060 10.1016/S1473-3099(14)70891-6

[19] Ding F, Zhang W, Wang L, Hu W, Soares Magalhaes RJ, Sun H, et al Epidemiologic features of severe fever with thrombocytopenia syndrome in China, 2011–2012. Clinical infectious diseases: an official publication of the Infectious Diseases Society of America. 2013;56(11):1682–3. Epub 2013/02/23. doi: 10.1093/cid/cit100 . 23429379 10.1093/cid/cit100

[20] Sun Y, Liang M, Qu J, Jin C, Zhang Q, Li J, et al Early diagnosis of novel SFTS bunyavirus infection by quantitative real-time RT-PCR assay. Journal of clinical virology: the official publication of the Pan American Society for Clinical Virology. 2012;53(1):48–53. Epub 2011/10/26. doi: 10.1016/j.jcv.2011.09.031 . 22024488 10.1016/j.jcv.2011.09.031

[21] Vynograd N. Natural foci diseases as a stable biological threat. Arch Immunol Ther Exp (Warsz). 2014;62(6):445–7. Epub 2014/10/20. doi: 10.1007/s00005-014-0316-8 . 25326726 10.1007/s00005-014-0316-8 PMC7079743

[22] Ding S, Niu G, Xu X, Li J, Zhang X, Yin H, et al Age is a critical risk factor for severe fever with thrombocytopenia syndrome. PloS one. 2014;9(11):e111736 Epub 2014/11/05. doi: 10.1371/journal.pone.0111736 . 25369237 10.1371/journal.pone.0111736 PMC4219771

[23] Li Z, Hu J, Bao C, Li P, Qi X, Qin Y, et al Seroprevalence of antibodies against SFTS virus infection in farmers and animals, Jiangsu, China. Journal of clinical virology: the official publication of the Pan American Society for Clinical Virology. 2014;60(3):185–9. Epub 2014/05/06. doi: 10.1016/j.jcv.2014.03.020 . 24793967 10.1016/j.jcv.2014.03.020

[24] Jin C, Liang M, Ning J, Gu W, Jiang H, Wu W, et al Pathogenesis of emerging severe fever with thrombocytopenia syndrome virus in C57/BL6 mouse model. Proceedings of the National Academy of Sciences of the United States of America. 2012;109(25):10053–8. Epub 2012/06/06. doi: 10.1073/pnas.1120246109 . 22665769 10.1073/pnas.1120246109 PMC3382536

[25] Li D. A highly pathogenic new bunyavirus emerged in China. Emerg Microbes Infect. 2013;2(1):e1 Epub 2013/01/01. doi: 10.1038/emi.2013.1 . 26038435 10.1038/emi.2013.1 PMC3630492

[26] Xu B, Liu L, Huang X, Ma H, Zhang Y, Du Y, et al Metagenomic analysis of fever, thrombocytopenia and leukopenia syndrome (FTLS) in Henan Province, China: discovery of a new bunyavirus. PLoS pathogens. 2011;7(11):e1002369 Epub 2011/11/25. doi: 10.1371/journal.ppat.1002369 . 22114553 10.1371/journal.ppat.1002369 PMC3219706

[27] Zhang YZ, Zhou DJ, Xiong Y, Chen XP, He YW, Sun Q, et al Hemorrhagic fever caused by a novel tick-borne Bunyavirus in Huaiyangshan, China. Zhonghua liu xing bing xue za zhi = Zhonghua liuxingbingxue zazhi. 2011;32(3):209–20. Epub 2011/04/05. . 21457654

[28] Jiao Y, Zeng X, Guo X, Qi X, Zhang X, Shi Z, et al Preparation and evaluation of recombinant severe fever with thrombocytopenia syndrome virus nucleocapsid protein for detection of total antibodies in human and animal sera by double-antigen sandwich enzyme-linked immunosorbent assay. J Clin Microbiol. 2012;50(2):372–7. Epub 2011/12/03. doi: 10.1128/JCM.01319-11 . 22135253 10.1128/JCM.01319-11 PMC3264160

[29] Liu Y, Huang XY, Du YH, Wang HF, Xu BL. [Survey on ticks and detection of new bunyavirus in some vect in the endemic areas of fever, thrombocytopenia and leukopenia syndrome (FTLS) in Henan province]. Zhonghua Yu Fang Yi Xue Za Zhi. 2012;46(6):500–4. Epub 2012/09/05. . 22943894

[30] Kang K, Tang XY, Xu BL, You AG, Huang XY, Du YH, et al [Analysis of the epidemic characteristics of fever and thrombocytopenia syndrome in Henan province, 2007–2011]. Zhonghua Yu Fang Yi Xue Za Zhi. 2012;46(2):106–9. Epub 2012/04/12. . 22490189

[31] Jiang XL, Wang XJ, Li JD, Ding SJ, Zhang QF, Qu J, et al [Isolation, identification and characterization of SFTS bunyavirus from ticks collected on the surface of domestic animals]. Bing Du Xue Bao. 2012;28(3):252–7. Epub 2012/07/07. . 22764528

[32] Jiao Y, Qi X, Liu D, Zeng X, Han Y, Guo X, et al Experimental and Natural Infections of Goats with Severe Fever with Thrombocytopenia Syndrome Virus: Evidence for Ticks as Viral Vector. PLoS neglected tropical diseases. 2015;9(10):e0004092 Epub 2015/10/21. doi: 10.1371/journal.pntd.0004092 . 26485390 10.1371/journal.pntd.0004092 PMC4618997

[33] Zhao L, Zhai S, Wen H, Cui F, Chi Y, Wang L, et al Severe fever with thrombocytopenia syndrome virus, Shandong Province, China. Emerging infectious diseases. 2012;18(6):963–5. Epub 2012/05/23. doi: 10.3201/eid1806.111345 . 22608264 10.3201/eid1806.111345 PMC3358154

[34] Niu G, Li J, Liang M, Jiang X, Jiang M, Yin H, et al Severe fever with thrombocytopenia syndrome virus among domesticated animals, China. Emerging infectious diseases. 2013;19(5):756–63. Epub 2013/05/08. doi: 10.3201/eid1905.120245 . 23648209 10.3201/eid1905.120245 PMC3647489

