# Supplementary material for: Preliminary fast diagnosis of severe fever with thrombocytopenia syndrome with clinical and epidemiological parameters
Source: PLoS One. 2017 Jul 5;12(7):e0180256. doi: 10.1371/journal.pone.0180256 (PMC5497983; doi:10.1371/journal.pone.0180256)
Supplement: S1 Appendix — (DOC) [file pone.0180256.s001.doc]

The questionnaire of suspected cases with SFTSV infection

Code No.□□□□□□□□□□□□□(see the appended code rule)

**1 Basic information**

1.1 Name: (please write down your parents’ names if you are under 14.)

1.2 Sex: ①male ②female

1.3 Race: ①Han ②others

1.4 Date of birth: (D) (M) (Y) (Without detailed date, please write down your exact age )

1.5 Occupation:

1. nursery children (2) scattered children (3)student (4)teacher (5)child-care worker (6)cook (7) waiter (8) shopping guide (9)tour guide (10) health care worker (11)cadres (12) laborer (13)migrant worker (14) farmer (15) forester (16)tea-picking worker (17)herdsman (18)hunter (19) butcher (20)Retiree (21)at home unemployed (22) unknown (23) other

1.6 Current Address:　 province　 　city(district/state )　 　county(city/district)　 　township(town/street)　 　village(neighborhood committees)　 　group(house number)

1.7 Contact Number:　 　 contacts:　 relationship:

1.8 ID number:

**2 Clinical process**
2.1 Date of onset: (D) (M) (Y)

2.2 Treatment

| Hospital times | Date | Medical institutions | Levels of  medical institutions | Diagnose | Medical records |
| --- | --- | --- | --- | --- | --- |
| The 1st time |  |  |  |  |  |
| The 2nd time |  |  |  |  |  |
| The 3rd time |  |  |  |  |  |
| The 4th time |  |  |  |  |  |

Note: Levels of medical institutions：(1)village (2) township (3)district (4)prefecture and above

2.3 Date of admission: (D) (M) (Y)

2.4 Admission number:

2.5 Admitting diagnosis:

2.6 Have you discharged from hospital : ①Yes ②No □

If you have:

2.6.1 Discharge diagnosis:

2.6.2 Date of discharge: (D) (M) (Y)

2.7 The patient’s condition under this survey:①recovered ②improved ③ worsen

④death□

2.8 Final outcome:①recovered ②death ③other

**3 Clinical manifestation**

3.1 Initial symptoms：

3.2 Systemic symptoms and signs：

3.2.1 [fever](javascript:void(0);) ①Yes T max: ℃ ②no □

3.2.2 chilly ①Yes ②No □

3.2.3 headache ①Yes ②No □

3.2.4 myalgia ①Yes ②No □

3.2.5 nephralgia ①Yes ②No □

3.2.6 conjunctival congestion ①Yes ②No □

3.2.7 skin petechiae or ecchymosis ①Yes ②No □

3.2.8 gum bleed ①Yes ②No □

3.2.9 anorexia ①slight ②anorexia ③No □

3.2.10 nausea ①Yes ②No □

3.2.11 vomiting ①Yes ②No □

3.2.12 haematemesis ①Yes ②No □

3.2.13 abdominal pain ①Yes ②No □

3.2.14 abdominal distension ①Yes ②No □

3.2.15 diarrhea ①Yes, times/day ②No □

3.2.16 stool property ①bloody stool　②melena　③watery stool ④other □

3.2.17 renal pain ①Yes ②No □

3.2.18 lymphadenopathy ①Yes ②No □

3.2.18.1 If you have, note the position, size and tenderness:

3.3 other:

**4 B**lood routine examination

| Time order | examination date (date  /month/year) | White blood cell count（10^9^/L） | Platelet count  （10^9^/L） | Neutrophil count（10^9^/L） | Lymphocyte count（10^9^/L） | Testing units |
| --- | --- | --- | --- | --- | --- | --- |
|  |  |  |  |  |  |  |
|  |  |  |  |  |  |  |
|  |  |  |  |  |  |  |

**5 Epidemiological investigation**

5.1 Types of residence ONE MONTH before the onset (multiple choice):

①Hill or mountain ② plain ③other________□

5.2 If you choose ②or③ in the 5.1 column, have you been to hills or mountains ONE MONTH before the onset?

①If you have, provide the specific locations(as detailed as possible)_________________ ②no ③ unkonw □

5.3 outdoor activities TWO WEEKS before the onset:

5.3.1 farming ①Yes ②No □

5.3.2 mowing ①Yes ②No □

5.3.3 hunting ①Yes ②No □

5.3.4 tea-picking ①Yes ②No □

5.3.5 grazing ①Yes ②No □

5.3.6 deforesting ①Yes ②No □

5.3.7 traveling to endemic areas ①Yes_____________ ②No □

5.3.8 other major activities _____________________________________

5.4 Are there ticks in your residence ONE MONTH before the onset?

①Yes ②No ③ unkonw □

5.5 Have you ever seen ticks ONE MONTH before the onset?

①Yes ②No ③ Don't know ticks □

5.6 Have you been bitten by ticks TWO WEEKS before the onset?

①Yes ②No ③ unkonw □

5.6.1 If you have, please write down the time/times:

①times:

②date of the first bite: (D) (M) (Y)

③date of the last bite: (D) (M) (Y)

5.6.2 Position of the bites (multiple choice):

①foot ②leg ③stomach ④back ⑤neck ⑥other □

5.7 Have your skin been damaged TWO WEEKS before the onset:①Yes ②No □

5.8Have you heard of patients who had similar symptoms (no close contact with them) in the neighborhood before the onset or not:①Yes ②No (jump to 5.9) □

5.8.1Basic information of similar patients that you’ve heard

| Name | Sex | Age | Address | Contact information |
| --- | --- | --- | --- | --- |
|  |  |  |  |  |
|  |  |  |  |  |
|  |  |  |  |  |

5.9 Whether you have close contact with the patients who had similar symptoms before the onset or not: ①Yes ②No（jump to 5.10) □

5.9.1 Basic information of similar patients who you’ve contacted

| Name | Sex | Age | Address | Relationship | Diagnosis | Way of contact | Contact information |
| --- | --- | --- | --- | --- | --- | --- | --- |
|  |  |  |  |  |  |  |  |
|  |  |  |  |  |  |  |  |
|  |  |  |  |  |  |  |  |

Note: Way of contact (multiple choice):① Direct contact with the patients’ blood

② Direct contact with the patients’ secretion /egesta

③ Treatment/nurse ④ Share the same room ⑤other contact (please note in the above table）□

5.10 Basic information of domestic animals：

①Yes (please fill out the following form) ②No ③ Unknown □

| Species of domestic animals | Whether you have close contact with animals within TWO WEEKS before the onset? | Are there ticks in domestic animals? |
| --- | --- | --- |
|  |  |  |
|  |  |  |
|  |  |  |

Note: Are there ticks in domestic animals: ①Yes ②No ③unknown □

5.11 Close contact with wild animals TWO WEEKS before the onset:

①Yes (please fill out the following form) ②No ③ unknown □

| Species of animals | Are there ticks in animals? | Notes |
| --- | --- | --- |
|  |  |  |
|  |  |  |
|  |  |  |

5.12 Have you found rats in house ONE MONTH before the onset:①Yes ②No ③ unkonwn □

**6 Summary：**

**7** **Specimen number (Code rule is enclosed.）:**

7.1 serum specimen:

7.1.1 serum number in acute phase: ;

7.1.2 serum number in recovery phase: ;

**8 Results of laboratory tests**

8.1 results of virus isolation: ①positive ②negative ③not test/not receive specimen□

8.2 results of nucleic acid detection: ①positive ②negative ③suspected ④not test/not receive specimen□

8.3 results of serological tests

|  | ELASA | | IFA | |
| --- | --- | --- | --- | --- |
|  | IgG | IgM | IgG | IgM |
| serum in acute phase |  |  |  |  |
| serum in recovery phase |  |  |  |  |

Note: please fill the blanks with：①positive ②negative ③not test/not receive specimen

Code rule: “year(2)- township-level region code(8) -serial number(3)”.

Region code is available in the direct reporting network system of CDC. For example, the code of the 12^th^ respondents in Shalang county, Wuhua district, Kunming city,Yunnan province is “10-53010220-012”. The serum numbers in acute phase and recovery phase respectively are J10-53010220-012 and H10-53010220-012, adding “J” and “H” before the code.

Signature of the investigator :

Date of the investigation: (D) (M) (Y)

Work unit:
